# Supplementary material for: Patterns of Intron Gain and Loss in Fungi
Source: PLoS Biol. 2004 Nov 30;2(12):e422. doi: 10.1371/journal.pbio.0020422 (PMC532390; doi:10.1371/journal.pbio.0020422)
Supplement: Table S1 — Also available at http://genes.mit.edu/NielsenEtAl/. (4.3 MB ZIP). [file pbio.0020422.st001.zip › NielsenEtAl/html/52.html]

AN6045.1.NCU07851.1.MG05281.1.FG04124.1


```
 CLUSTAL W (1.82) Multiple Sequence Alignments - Introns Inserted


Sequence 1: MG05281.1	266 aa
Sequence 2: FG04124.1	248 aa
Sequence 3: NCU07851.1	270 aa
Sequence 4: AN6045.1	247 aa
Alignment Length: 270 aa
Number Identitical Residues: 103 aa
Alignment Score (without introns) 5477


MG05281.1 	-MGVNHPFQ0TLFAVHMTCDSCVKDVSDAVQKLGG~ITKVDANLKDQLVSIEGTA1APSA
NCU07851.1	MASITTPFK0TLFAVPMHCESCVNDVSGALYKLPG~INKVEANLKDQLLTIEGTA~APSA
FG04124.1 	-MTVDNSFE0TLFAVPLSCDGCIKAVSDSLYKLGG~IKNVEGNLKDQLVSVKGTA1APSA
AN6045.1  	---MIEPFQ0TTFAVPMTCDGCVKDISQALHKVEG1ITKVEANLKDQLVFIEGTA~PPSS
          	   :  .*: * *** : *:.*:: :* :: *: * *.:*:.******: ::*** .**:

MG05281.1 	IVDAIQATGRDAILRGSGSSDS1AAVSILETYQHLSVVETHEERAKRERQVRGLARMVQV
NCU07851.1	IVNAIQSTGRDAILRGSGDSQG~AAVSILETYHKPTLDKITPAPGASDRMVRGLARMVQV
FG04124.1 	IVEAIQATGRDAILRGSGASNS1AAVSILETFEDP-VDGLYEEPS---RDVRGLARMVQV
AN6045.1  	IVTAIQNTGRDAILRGTGASNN1SAVCILETHATS-------VPN----KIRGLARMVQV
          	** *** *********:* *:. :**.****.                  :*********

MG05281.1 	SPEVTLVDLTVRGVSPGSYQVTIREYGNLTEGVESAGPVWAGSDRPATNPSANGAA---K
NCU07851.1	NNSTTLVDLTLRGIAPGTYRATIREFGDLARGVESAGPVWSGTATLTADTKAQISGDPNA
FG04124.1 	SSGRTLVDLSIRGVSPGKYRASIRAYGDLKNGATSTGPVWSGEDK--------------K
AN6045.1  	SSNMTLVDLTINGLAPGKYWATVREAGDISKGAESTGGIWEAVKAKLQGANAQK-----E
          	.   *****::.*::**.* .::*  *:: .*. *:* :* .      ...:.       

MG05281.1 	PRGFLGTVQVGKEGHGSVFMEASFQVWEIIGHAMIVSPHDEAASGGKILKNDADTVAGIV
NCU07851.1	PRGVLGTVEINKDGRGSVFLSHPFQIWEVIGHAFAVAPVSVDESAGSTLENDENTVVGVI
FG04124.1 	LRGDLGLVEVGKDGRGASFVDHEFQIWEVIGHAMVLTRQEEKAEP---LKNDKDTVVGII
AN6045.1  	PRGIFGSVEVDAKGRGNVFLDRPVAIWELIGRSMVVSKSTEGPFR----REDSNTLVGVI
          	 ** :* *::. .*:*  *:.  . :**:**::: ::            .:* :*:.*::

MG05281.1 	ARSAGVWGNDKTVCACSGKTLWEERKDEIQKGMF
NCU07851.1	ARSAGVWDNDKTVCSCTGKTLWEERKDEVEKGML
FG04124.1 	ARSAGMWDNDKTVCSCTGKTLWEERKDEVKKGML
AN6045.1  	ARSAGVWDNDKTVCSCSGKNVWQERQEQVAQGML
          	*****:*.******:*:**.:*:**:::: :**:
```
